# Executive Functions in Predicting Weight Loss and Obesity Indicators: A Meta-Analysis

**DOI:** 10.3389/fpsyg.2020.604113

**Published:** 2021-01-28

**Authors:** Zhongquan Du, Jingjing Li, Jiaai Huang, Jing Ma, Xiaoyu Xu, Rong Zou, Xia Xu

**Affiliations:** ^1^Graduate School, Wuhan Sports University, Wuhan, China; ^2^College of Health Science, Wuhan Sport University, Wuhan, China; ^3^Hubei Key Laboratory of Sport Training and Monitoring, Wuhan Sports University, Wuhan, China

**Keywords:** executive function, obesity, weight loss, overweight, cognitive function, cognitive training

## Abstract

While previous studies have suggested that there exists a relationship between obesity and executive function (EF), the mechanisms and causal relationship between them remain unclear. There are important clinical implications of determining whether EF can predict and treat obesity. We conducted a multilevel meta-analysis of randomized controlled trials (RCTs) and longitudinal studies. Specifically, we investigate (a) whether EF interventions have an effect on weight loss, (b) whether baseline EF can be a predictor of future weight loss through obesity intervention, and (c) whether early-life EF can predict future weight loss. Eight RCTs and 17 longitudinal studies with a total of 11,393 participants were identified. We found that (a) EF interventions may not have an effect on weight loss, (b) baseline inhibition (β = 0.259, *p* = 0.03) and delay discounting (β = −0.17, *p* = 0.04) significantly predict future weight loss through obesity intervention, (c) age (*F* = 13.666, *p* = 0.005) moderates the relationship between working memory and weight loss through intervention, but not weight status, type of intervention, and percentage of female, and (d) early life inhibition (β = 0.185, *p* = 0.07) is a marginally significant predictor of future weight loss. Our results seem to support the assumption that the relationship between EF and obesity is not direct, and a higher-order factor, such as genes, may link obesity and EF. Building on the preliminary findings, further studies focusing on EF and obesity are needed in the future.

## Introduction

Obesity is a worldwide health problem. According to the World Health Organization, 39% of adults were overweight, and 13% were obese in 2016 (World Health Organization, 2018). Being obese is a risk factor for many psychological and physiological diseases, and there is much evidence showing that excess weight is associated with type 2 diabetes (Schwartz and Porte, 2005), cardiovascular diseases (Nakamura et al., 2014), cancer (Calle and Kaaks, 2004), depression (Luppino et al., 2010), and anxiety (Gariepy et al., 2010). Furthermore, obese individuals often experience discrimination (Tomiyama, 2014). Obese individuals may have lower socioeconomic status positions (Wang and Beydoun, 2007), and obese daughters receive less financial support from their parents than their lower body mass index (BMI) sisters (Crandall, 1995). Since obesity has nearly tripled since 1975 (World Health Organization, 2018), the public should pay more attention to obesity and weight loss.

In recent years, a growing number of researchers have linked obesity to executive functions (EFs). A number of studies found that obese and overweight individuals had poor EF performances compared to their counterparts (Hayes et al., 2018; Yang et al., 2018; Favieri et al., 2019; Mamrot and Hanc, 2019). Successful weight loss through intervention has been associated with improvements in EFs (Veronese et al., 2017). Moreover, cognitive training and neuromodulation strategies are seen as promising ways to overcome excess weight (Forcano et al., 2018).

Executive function (EF), also referred to as executive control or cognitive control, is an umbrella term involving interrelated higher-order neurocognitive mental processes that enable goal-directed behaviors (Banich, 2009; Diamond, 2013). There are two types of EF: cool cognitive EF and hot affective EF (Prencipe et al., 2011; Zelazo and Carlson, 2012). Cool EF, such as inhibitory control, working memory, and attentional shifting, refers to cognitive functions that engage in solving abstract and novel problems without the involvement of affect. In contrast, hot EF, such as delay discounting and affective decision making, refers to cognitive functions that involve affect when solving problems. According to Diamond (2013), there are three core EFs: (1) inhibition is the ability to control one's attention, thought, behavior, and emotion, including self-control, behavior control, selective attention, and cognitive inhibition; (2) working memory is the ability to update information and monitor ongoing action and stimuli; and (3) cognitive flexibility is the ability to shift attention, thought, perspective, and mindset in response to environmental conditions. Higher-order EFs such as planning and problem-solving are built up from these three core EFs.

Previous neuroimaging studies found that EF may mainly rely on the prefrontal cortex (Moriguchi and Hiraki, 2013). For instance, inhibition may associate with the inferior frontal cortex, dorsolateral prefrontal cortex, and orbital frontal cortex in the view of the localizationist (Aron et al., 2014). Although neurophysiological studies found that the persistent discharges widespread occurred in cortical and subcortical regions, such as the primary visual and posterior parietal cortices, when participants were engaged in working memory task (Constantinidis and Procyk, 2004; Pasternak and Greenlee, 2005), the prefrontal cortex is still one of the most important regions for working memory (Riley and Christos, 2015). Cognitive flexibility is a relatively advanced cognitive process that involves many brain regions such as basal ganglia, anterior cingulate cortex, and posterior parietal cortex, and undoubtedly, the prefrontal cortex is an important part of it (Leber et al., 2008). A functional magnetic resonance imaging study (Adleman et al., 2002) found a significantly different brain activity in the prefrontal region among children (age 7–11), adolescents (age 12–16), and young adults (age 18–22) when engaged in inhibition on the Stroop color–word task, the result of which was consistent with the idea that EFs continue to develop through childhood and adulthood. However, the developmental trajectories of different EF domains are different (Best and Miller, 2010). Inhibition appears to rapidly develop during the preschool years and shows less change later on, while working memory and cognitive flexibility develop at a relatively stable rate. One interpretation of such differences is that the brain regions supporting EFs are different (O'Connor, 2002).

### The Role of EF on Obesity and Weight Loss

EF plays an important role in our lifestyle. Lower EF has been associated with higher levels of unhealthy food consumption (Pieper and Laugero, 2013), more sedentary behavior (Wirt et al., 2015), less physical activity (Riggs et al., 2010), and lower levels of fruit and vegetable consumption (Zhou et al., 2015). EF deficits may lead to obesity through obesogenic habits.

A dual-process model suggested that our eating behaviors are guided by two distinct but associated processes: automatic and reflective processes (Hofmann et al., 2009). Automatic processes associate food-related cues with rewarding effects so that food-related cues can automatically draw one's attention, evoke approach responses, and then influence eating behaviors. Automatic processes occur without cognitive resources and may overwhelm conscious intentions. The reflective processes, also named the controlled processes, are top-down, conscious processes that elicit behaviors as a consequence of decision making and are closely linked to EF. People with a weak reflective system could easily be driven by the automatic system, which would lead them to choose more high-energy-dense food and therefore be obese.

What causes an individual to become obese is that they consume more energy than they expend (Spiegelman and Flier, 2001). Our understanding of the mechanisms of obesity has been improved over decades of research. There are three factors that are related to obesity. The first is homeostasis, which is the maintenance of energy balance by regulating internal physiological processes (Narayanaswami and Dwoskin, 2017). When an individual is objectively hungry or full, the body will spontaneously release signals to maintain energy balance. For example, when your body is hungry, the homeostasis system will make you feel hungry and urge you to eat. The second is reward system. Food itself is a natural reward that refers to a person's natural desire for food (Berridge, 1996). Certain foods, especially those high in sugar and fat, are effective rewards for promoting eating even when there is no energy requirement (Berridge and Robinson, 2003). This is also the reflection process in the dual-process model mentioned earlier. The third is executive function. Homeostasis and reward systems are innate and spontaneous, not controlled by consciousness, while executive function depends on conscious effort. Executive function determines whether to eat and allows us to refuse to eat food in the face of food temptation or hunger. In this way, EF may be able to compete with homeostatic and reward systems, thereby reducing overall energy intake. Therefore, for obese people, better EF may help them regulate their eating behaviors to achieve the goal of weight loss.

Several systematic reviews have investigated the effect of EF on obesity and weight loss (Hayes et al., 2018; Favieri et al., 2019; Mamrot and Hanc, 2019). Hayes et al. (2018) found that compared to normal-weight individuals, obese children showed poorer EF performances. They came to the preliminary conclusion that behavioral weight loss interventions may improve EF performance, which in turn improved the outcomes of interventions. The existence of a relationship between EF and obesity has been confirmed (Favieri et al., 2019). EF and obesity were dependent (Mamrot and Hanc, 2019); however, the mechanisms of them could not be determined (Favieri et al., 2019), and whether EF can be a predictor of obesity remained unknown (Mamrot and Hanc, 2019).

In sum, these researchers found an association between EF and obesity, but the causal relationship could not be determined. All of these studies were not meta-analyses that quantitatively synthesized the data, and they either (a) considered only a specific age stage (Mamrot and Hanc, 2019), (b) aimed only at whether EF influenced obese and overweight individuals (Hayes et al., 2018; Favieri et al., 2019), and (c) did not exclusively focus on the influence of EF (Forcano et al., 2018; Jones et al., 2018). Although many researchers hold the view that EF may have an influence on obesity and weight loss, there is no strong evidence of such a relationship, and the mechanism is not clear. Determining whether EF can predict and treat obesity has important clinical implications.

#### EF Intervention on Obesity and Weight Loss

Because cognition is important in eating behavior and it is controlled by consciousness, cognitive training has recently gained increasing attention. Previous studies found that repeated cognitive training on EF tasks can improve EF capacity, which makes it possible for EF interventions to become an effective way to treat obesity (Manuel et al., 2013; Berkman et al., 2014). EF can be trained by gradually increasing the difficulty of the EF task, and participants' performances on the EF task are indicators of their EF level. There are also some other cognitive training interventions, such as episodic future thinking (Atance and O'Neill, 2001), approach/avoidance training (Schonberg et al., 2014), attention bias modification (Kemps et al., 2014), and implementation intentions (Armitage, 2004). Several systematic reviews of whether these interventions affect obesity and weight loss have been conducted. However, the results of these systematic reviews were different. Yang et al. (2019) found that, with the exception of inhibition training, other types of cognitive training made no contribution to weight loss. However, Forcano et al. (2018) found that in addition to inhibition training, implementation intentions showed promising results regarding weight loss in people with obesity/overweight, and attention bias modification had an effect in normal-weight participants, while other types of cognitive training were ineffective. The effects of cognitive training need to be further explored.

On the one hand, these cognitive trainings are all related to EF. On the other hand, they involve various cognitive processes in addition to EF, which may make the issue more complicated. Thus, it would be helpful to merely consider the effect of EF intervention. However, randomized controlled trials (RCTs) investigating the effect of EF interventions have shown inconsistent results. While some studies claimed that weight loss intervention through improving EFs significantly reduced participants' BMI (Allom and Mullan, 2015; Raman et al., 2018; Galindo Muñoz et al., 2019), other studies did not find such a pattern (Houben et al., 2016; Dassen et al., 2018b). A meta-analysis using RCTs that may reveal a causal inference is needed to assess these inconsistencies.

#### EFs as Predictors of Weight Loss

Current weight loss programs focus on reducing energy intake and increasing energy expenditure. The effects of obesity interventions vary from person to person. In bariatric surgery, for example, most patients achieve their weight loss goals after surgery; however, there are still a large number of people who fail to achieve their weight loss goals and even regain the weight a few years after surgery (Karmali et al., 2013; Maleckas et al., 2016). Therefore, it is important to understand the individual factors that influence obesity intervention. The physiological and behavioral factors are the two types most likely to influence obesity intervention outcomes. For example, Wildes et al. (2010) found that children with binge eating lost less weight through family-based obesity intervention. Augustijn et al. (2018) found that balance skills significantly predict weight loss in a 5-month treatment program. Given the close link between EF and obesity, EF is likely to be a strong predictor of obesity intervention outcomes.

EFs can facilitate goal-directed activities and eating behaviors, which may have an impact on obesity interventions (Appelhans et al., 2016). For example, cognitive function is associated with adherence to bariatric postoperative guidelines (Beth Spitznagel et al., 2013), which may be the reason that bariatric patients with poor long-term weight loss have poorer inhibitory control than patients who lose their weight successfully (Hogenkamp et al., 2015). Besides, as the mechanism we discussed above, the impact of EF on eating behavior occurs all the time, it is reasonable to believe that different baseline levels of EF may affect obesity treatment outcomes. Determining the predictive factors of weight loss is important for preventing and treating obesity.

Inhibition, delay discounting, working memory, cognitive flexibility, planning, and general EF are considered to be predictors of obesity intervention outcomes. However, inconsistencies also occurred in longitudinal studies investigating whether better baseline EF can predict more weight loss through obesity interventions. Butryn et al. (2019) found that baseline EF measured by the tower task component of the Delis–Kaplan Executive Function System significantly predicted weight loss through 6-month standard behavior treatment. A study of 82 obese adults from an obesity intervention center found that baseline inhibition, shifting, and delay discounting, measured by the stop-signal task, trail-making test, and monetary choice questionnaire, respectively, were not predictors of weight loss, while better working memory and self-reported inhibition measured by the 2-back test and Behavioral Rating Inventory of Executive Functioning-Adult Version, respectively, were predictive of more weight loss across 6-month behavioral treatment.

The above discussion focused on whether EF can predict obesity interventions outcomes. However, whether EF can predict obesity or weight loss under natural conditions is also an important issue. Girls with poorer inhibition at age 7 have higher BMI and more weight gain at ages 9, 11, 13, and 15 (Anzman and Birch, 2009). The ability to delay gratification of preschoolers can even predict their BMI 30 years later (Schlam et al., 2013). Although there is relatively less concern in this area, this is important for understanding the effect of EFs on obesity and weight loss. A meta-analysis is needed to summarize these findings.

### The Aim of the Present Study

Although many researchers agree that EF can influence obesity and weight loss, the causal relationship between them could not be determined. If EF does affect obesity, a more effective obesity intervention could be proposed. There are important clinical implications of determining whether EF can treat obesity. However, as discussed above, RCTs investigating the effect of EF interventions have shown inconsistent results (Verbeken et al., 2013; Allom and Mullan, 2015; Houben et al., 2016; Galindo Muñoz et al., 2019). A meta-analysis using RCTs that may reveal a causal inference is needed to assess these inconsistencies. At the same time, it is also important to investigate the predictive effect of EF on obesity and weight loss through obesity interventions and natural conditions. If EF does predict obesity and weight loss, we may take precautions against obesity. To our knowledge, this is the first meta-analysis to investigate whether EFs have an impact on obesity and weight loss. We focused on all ages and all weight statuses (normal weight, overweight, and obesity) to obtain a general conclusion. The aim of this study is to determine (a) whether EF interventions have an effect on weight loss, (b) whether better baseline EF can predict greater weight loss through weight loss intervention, and (c) whether early life EF can predict future weight loss.

In addition, we examined some potential moderating factors. As discussed earlier, the effect of cognitive training on weight loss varies by weight statuses, and the levels of EFs differ by age. Age and weight status may moderate the relationship between EF and obesity. In longitudinal studies, surgical interventions are invasive while non-surgical interventions are not (Puzziferri et al., 2014). There is a large difference between surgical and non-surgical interventions. In some studies, the percentage of female participants was 100%, which may have influenced the results. Therefore, we examined age, weight status, type of intervention, and percentage of female participants as potential moderators of EF and weight loss.

## Methods

This systematic review was conducted according to the PRISMA statement (Liberati et al., 2009; Moher et al., 2009). The PROSPERO registration number is CRD42020177212.

### Searching Strategies and Study Selection

**Figure 1:**
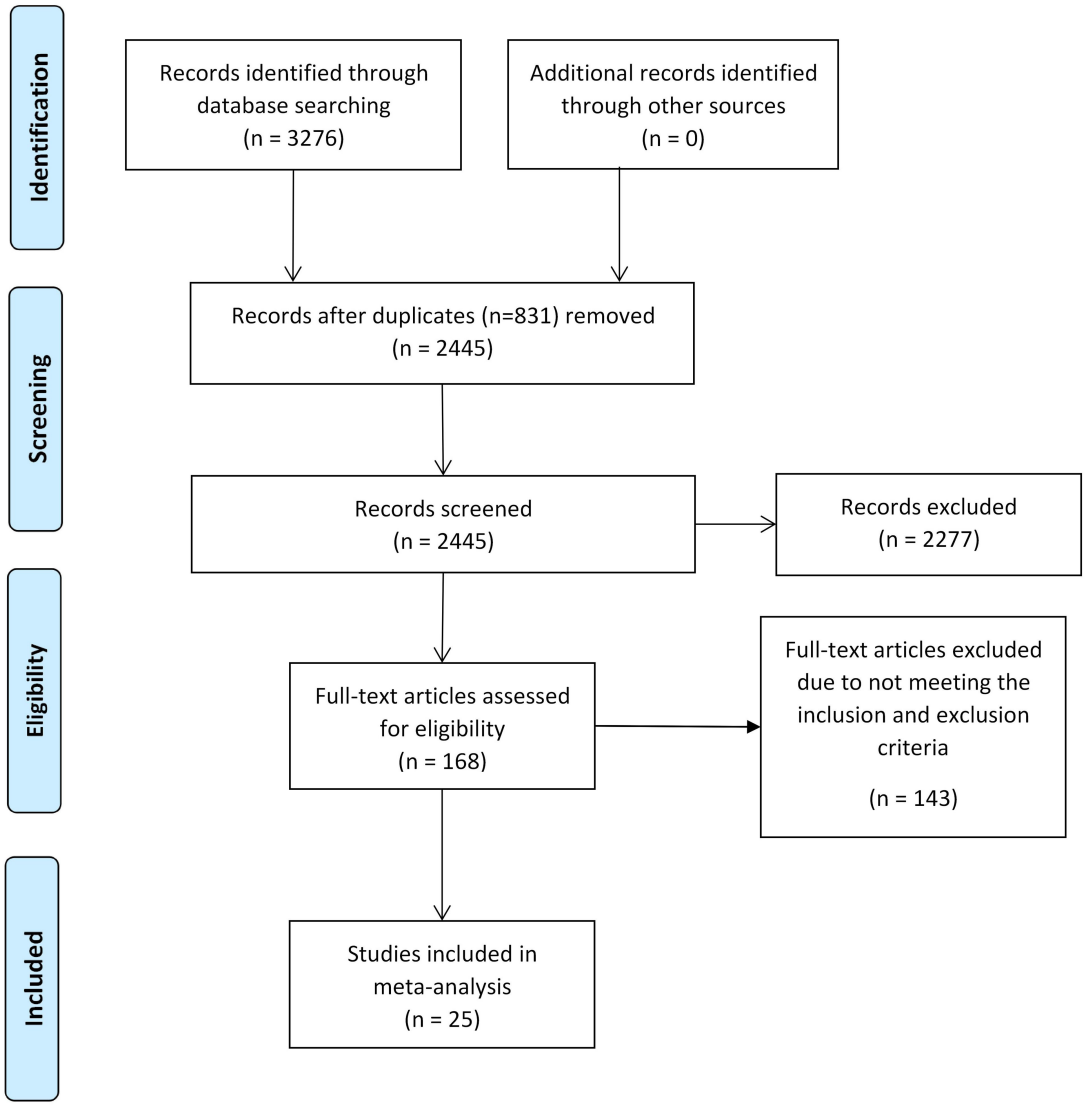
Flow diagram illustrating the process of our review, screening, and article selections.

Two investigators (Z.D., J.M.) independently conducted the literature search. Four databases, PubMed, Web of Science, Scopus, and psycINFO, were utilized to obtain the studies for meta-analysis on January 11, 2020. The searching strategies are shown in Table 1. To ensure that we found as many studies as possible, references in relevant studies were also reviewed. The detailed selection procedure is shown in Figure 1.

**Table 1:** Searching strategies.

**Data base**	**Searching terms**
Web of science and Scopus	Topic = (“executive function” OR “executive control”) AND Topic = (“obesity” OR “obese” OR “overweight” OR “weight loss”) NOT Topic = (“rats” OR “birds” OR “monkey” OR “primate” OR “mice”)
psycINFO	mjsub (“obesity” OR “obese” OR “overweight” OR “weight loss”) AND mjsub (“executive function” OR “executive control” OR “inhibition” OR “delay discounting” OR “working memory” OR “shifting” OR “switching” OR “cognitive flexibility” OR “planning”) NOT mjsub (“rats” OR “birds” OR “monkey” OR “primate” OR “mice”)
PubMed	(((“obesity” OR “obese” OR “overweight” OR “weight loss”[MeSH Major Topic]) AND (“executive function” OR “executive control” OR “delay discounting” OR “inhibitory control” OR “working memory” OR “shifting” OR “switching” OR “cognitive flexibility” [MeSH Major Topic])) AND (Englishi[Language])) AND (humans[MeSH Terms])

The following inclusion criteria were used: (a) studies written in English, (b) participants in studies were human, and (c) studies were published in peer-reviewed journals. For the RCTs investigating whether EF interventions were effective, they (a) should include at least one control group, (b) should aim at weight loss through improving EF, and (c) should report participants' baseline and followed-up measures or changes in BMI or other indicators of weight status. For the longitudinal studies investigating whether EF predicts more weight loss through intervention and predicts future weight loss, (a) linear regression should be employed, (b) baseline EF should be used to predict future BMI changes or changes in other indicators of weight status, and (c) standard error of standardized regression coefficient beta should be reported or could be calculated.

We excluded studies if they (a) used secondary data whose first-hand data were included in the meta-analysis, (b) were not empirical studies, (c) lacked important data that were necessary for the meta-analysis, (d) included participants with any significant or chronic condition known to affect cognitive status (e.g., depression, cancer), and (e) included subjects with type 2 diabetes, cardiovascular event antecedents, and other physiological diseases that may influence their weight status.

### Quality Assessment

The quality of the study was independently assessed by two reviewers (Z.D., J.M.), and any discrepancies were mediated by the third reviewer (X.X.). For the RCTs, the quality was assessed through Jadad's scale (Jadad et al., 1996), which is one of the most commonly used quality assessment tools for RCTs by researchers with good reliability (Clark et al., 1999). We did not consider blinding as all RCTs were single-blind trials and the same method was used to control the placebo effect; therefore, there was a total of three points in terms of randomization and dropout.

Currently, there are few tools available to evaluate longitudinal observational studies; The Strengthening the Reporting of Observational Studies in Epidemiology (STROBE) (von Elm et al., 2008) guidelines were one of the quality assessment tools to assess longitudinal studies, and it has been used in recent systematic reviews (Scales and Dahm, 2008; Ricci-Cabello et al., 2010). STROBE contains a total of 22 items, and 9 of them relevant to the methodology were selected to assess the quality.

### Data Extraction

Data extraction was also independently conducted by two reviewers (Z.D., J.M.), and disagreement was mediated by the third reviewer (X.X.). Details of the EF intervention, participant characteristics, baseline weight status, and eligibility criteria of the participants were extracted from the RCTs. For the longitudinal studies, the participant characteristics, baseline BMI, time between baseline, and endpoint, EF of interested and their measurements were identified.

### Statistical Analysis

As some studies involved multiple outcomes and the results are not independent of each other, a three-level meta-analytic model followed by Assink and Wibbelink (2016) was conducted. This model takes into account three kinds of variance: (a) sampling variance (level 1), which is the variance in the observed effects, (b) within-study variance (level 2), which is the variance for multiple outcomes in the same studies, and (c) between-study variance (level 3), which is the variance in effects between different studies. Significant variance in within-study or between-study indicates heterogeneity between studies. Restricted maximum likelihood estimation (REML) was used to reduce the bias of variance estimates (Van den Noortgate et al., 2015). As the participants in these studies varied in age, country, and economic status, a random-effect model was employed.

When combining effect sizes, Hedges' *g* was used to estimate the pooled effect size for the RCTs, as it is a relatively unbiased estimation. For longitudinal studies, the standardized regression coefficient β was used as the effect size (Kim, 2011; Bowman, 2012). One RCT study (Galindo Muñoz et al., 2019) did not provide the posttest BMI standard deviation (SD), and we used baseline SD to estimate it (Higgins and Green, 2011). The standard error of β was calculated by β/t or SE × S_X_/S_Y_, where t is the result of the significance test, SE is the standard error of the unstandardized regression coefficient, S_X_ is the variance in the independent variable, and S_Y_ is the variance in the dependent variable. For studies that did not provide t values, S_X_ or S_Y_, the *P*-value was used to calculate t, and if the *P*-value is less than a 0.05 threshold, a conservative estimate of this threshold minus 0.01 was taken as the *P*-value.

For the RCTs, a positive effect size means that compared to the control condition, the EF intervention was more effective. For the longitudinal studies, a positive effect size means that better EF predicted more weight loss. If the number of studies was > 3, a subgroup analysis was conducted in terms of age, type of intervention, percentage of female participants, and weight status to explore the moderating effects. Q and I-squared statistics were used to measure the study heterogeneity. Publication bias is an important issue in the meta-analysis, which may affect the accuracy of results. Publication bias refers to the fact that studies with insignificant results are less likely to be published than those with significant results (Rosenthal, 1995), which leads to bias in the literature included in the meta-analysis. Funnel plots and Egger's test (Egger et al., 1997) were used to assess publication bias. The funnel plot is a qualitative method to observe whether there is publication bias. If the funnel plot is observed to be symmetrical, there is no publication bias. As the observation of funnel plots is highly subjective, Egger's test can objectively measure whether the funnel plots are symmetrical or not. If the test results are significant, it means that the funnel plot is statistically asymmetric and there is missing data. In the case of significant Egger's test results, the trim-and-fill method (Duval and Tweedie, 2000) was employed to correct the asymmetry of funnel plots caused by publication bias. The multilevel meta-analysis was conducted in R (version 3.6.3) with the metafor package.

## Results

### Study Characteristics

There were totally 11,393 participants included in our meta-analysis. The participants' characteristics are summarized in Table 2. Eight studies (Verbeken et al., 2013; Allom and Mullan, 2015; Stice et al., 2015, 2017; Houben et al., 2016; Dassen et al., 2018b; Raman et al., 2018; Galindo Muñoz et al., 2019) with 716 participants were found, and one study (Allom and Mullan, 2015) included two independent substudies, for a total of 9 RCTs. Seven RCTs solely focused on one specific EF, and in particular, 4 of them focused on inhibition, 2 of them focused on working memory, and 1 focused on cognitive flexibility. Four RCTs (Verbeken et al., 2013; Dassen et al., 2018b; Raman et al., 2018; Galindo Muñoz et al., 2019) employed other weight loss interventions addition to the EF intervention. Regarding quality, 5 RCTs scored 3 points, 2 RCTs scored 2 points, and only 1 RCT scored 1 point. More details about the studies are presented in Table 3.

**Table 2:** Participants characteristics.

**Type of analysis**	**Executive function**	***N***	**Mean BMI (kg/m^**2**^)**	**Age**	**Weight status**	**Female (%)**
Meta-analysis of RCTs	Inhibition	355	24.95	22.13	Normal weight Overweight Obesity	77.62%
	Working memory	141	31.04	44.04	Overweight Obesity	74.47%
	Cognitive flexibility	80	39.78	41.36	Overweight Obesity	86.00%
	General EF	140	NA	29.41	Overweight Obesity	67.68%
Meta-analysis of longitudinal studies investigating whether baseline EFs can predict greater weight loss through intervention	Inhibition	220	NA	31.79	Overweight Obesity	67.55%
	Working memory	202	42.96	42.55	Obesity	74.77%
	Cognitive flexibility	245	41.24	40.23	Obesity	66.15%
	General EF	228	43.84	39.6	Obesity	80.29%
	Delay discounting	376	NA	17.54	Normal weight Overweight Obesity	64.91%
	Planning	56	36.03	26.53	Obesity	60.76%
Meta-analysis of longitudinal studies investigating whether early EFs can predict future weight loss	Inhibition	7,374	NA	6.35	NA	49.84%
	General EF	2,450	NA	10	Normal weight Overweight Obesity	100%
	Delay of gratification	164	NA	4	NA	58%

*NA, not available; BMI, body mass index*.

**Table 3:** RCTs investigating whether EF interventions are effective.

**References**	**Intervention**	**Executive functions**	**Participants**	**Eligibility criteria**	**Weight status**	**Quality** **score**
Allom and Mullan (2015) **(1)**	Online stop-signal task training intervention: 10 sessions, 192 trials per session, 50% unhealthy food pictures. Training: inhibit 50% of unhealthy food pictures. Control 1: inhibit 25% of unhealthy food pictures and 25% of healthy food pictures. Control 2: no inhibition. Duration: 12 days. Assessment: baseline, posttest	Inhibition	N: Training: 29, Control 1: 25, Control 2: 28. Mean age: 20.43. Female (%): 80.5.	Inclusion: have the intention to change dietary behavior, not color blind. Exclusion: have a current or prior eating disorder diagnosis.	Mean BMI: Training: 22.21, Control 1: 22.78, Control 2: 22.90.	3
Allom and Mullan (2015) **(2)**	Online stop-signal task training intervention: 10 sessions, 192 trails per session, 50% unhealthy food pictures. Training: inhibit 50% of unhealthy food pictures. Control 1: inhibit 25% of unhealthy food pictures and 25% of healthy food pictures. Control 2: no inhibition. Duration: 12 days. Assessment: baseline, posttest, 1 week after training.	Inhibition	N: Training: 27, Control 1: 26, Control 2: 25. Mean age: 22.97. Female (%): 78.2.	Inclusion: have the intention to change dietary behavior, not color blind. Exclusion: have a current or prior eating disorder diagnosis.	Mean BMI: Training: 23.11, Control 1: 23.01, Control 2: 23.21.	3
Dassen et al. (2018b)	Working memory training: 25 sessions, 90 trials per session, visuospatial WM task, backward digit span task and object memory task, 30 trials per task. Training: difficulty level of the tasks was automatically adjusted on a trial-by-trial basis Control: difficulty level of the tasks was held constant at a basic level. Duration: 25 days. Assessment: baseline, posttest, 1 and 6 months after training.	Working memory	N: Training: 51, Control: 40. Mean age: 47.97. Female (%): 74.7.	Inclusion: age: 18–60, overweight, have motivation to put in effort to achieve weight loss. Exclusion: in treatment for an eating disorder or in the trajectory of bariatric surgery.	Overweight; mean BMI: Training: 30.96, Control: 30.49.	2
Galindo Muñoz et al. (2019)	Cognitive training intervention: 12 sessions Training: 12 different practice exercises per session, cognitive training was conducted via the video game *Brain Exercise^*TM*^*. Control: cognitive-behavior therapies and nutritional education. Duration: 12 weeks. Assessment: baseline, posttest.	Working memory Inhibition Cognitive flexibility Planning	N: Training: 48, Control: 48. Mean age: Training: 31.18, Control: 31.71. Female (%): 74.	Inclusion: BMI ≥ 27. Exclusion: subjects with type 2 diabetes, cardiovascular event antecedents, any significant or chronic condition known to affect cognitive status, subjects using some sort of pharmacological treatment that could affect body weight or to be on a dietary treatment or diet within the previous 6 months before.	Obesity, overweight; mean BMI: Training: 31.18, Control: 31.71. Body fat (%): Training: 37.04, Control: 37.62. Waist circumference: Training: 101.9, Control: 101.4.	3
Houben et al. (2016)	Working memory training: 20–25 sessions, 90 trails per session, visuospatial WM task, backward digit span task and object memory task, 30 trails per task. Training: difficulty level of the tasks was automatically adjusted on a trial-by-trial basis Control: difficulty level of the tasks was held constant on a basic. Duration: 25 days. Assessment: baseline, posttest, 1 month.	Working memory	N: Training: 24, Control: 26. Mean age: Training: 36.08, Control: 37.62. Female (%): Training: 79.2, Control: 69.2.	Inclusion: age: 18–65, BMI higher than 25	Obesity, overweight; mean BMI: Training: 31.76, Control: 31.38.	2
Raman et al. (2018)	Cognitive remediation therapy: 8 sessions Training: mental exercises aimed at improving cognitive strategies, thinking skills and information processing through practice. The intervention was conducted face-to-face. Control: no treatment. Duration: 9–11 weeks. Assessment: baseline, posttest, 3 months.	Cognitive flexibility	N: Training: 38, Control: 42. Mean age: Training: 40.6, Control: 42.2. Female (%): NA.	Inclusion: BMI ≥30, age: 18–55 years, current weight under 180 kg, having completed 10 years of education in English. Exclusion: had a history of psychosis, head injury, neurological disorder; unable to complete the testing; on regular sedative or stimulant medication; and/or report regular substance use or abuse.	Obesity; mean BMI: Training: 40.3, Control: 39.2.	3
Stice et al. (2015)	Cognitive reappraisal obesity prevention program: Training: learn and practice how to use cognitive reappraisals to reduce desire for and intake of unhealthy foods, 7 1-h weekly. Control 1: change the dietary intake and physical activity. Control 2: view *Weight of the World*, a 51-min documentary on obesity. Duration: 7 weeks. Assessment: baseline, posttest, 6 month.	Inhibition	N: Training: 25, Control 1:61, Control 2: 62. Mean age: 19.3. Female (%): 72.	Inclusion: 1st-year college students. Exclusion: current DSM-IV anorexia nervosa, bulimia nervosa, or binge eating disorder.	Obesity, overweight, normal weight; mean BMI: 23.5. Body fat (%): Training: 26.21, Control 1: 23.7, Control 2: 26.54.	2
Stice et al. (2017)	Response training intervention: 4 sessions, 5 tasks per session: stop-signal training (320 trials), go/no-go training (300 trials), respond-signal training (352 trials), dot-probe training (320 trials), Visual-search training (120 trials). Training: 80 high-calorie food and 80 low-calorie food images, inhibit 100% high-calorie food. Control: 80 images of birds and 80 images of flowers. Duration: 4 weeks. Assessment: baseline, posttest, 6 months.	Inhibition	N: Training: 23, Control: 24. Mean age: Training: 32.8, Control: 32.4. Female (%): 91.	Inclusion: BMI ≥25. Exclusion: current DSM-IV anorexia nervosa, bulimia nervosa, or binge eating disorder.	Obesity, overweight; Mean body fat (%): Training: 46.89, Control: 43.10; mean BMI: Training: 38.46, Control: 35.	1
Verbeken et al. (2013)	Executive functioning training with game elements: Training: 25 sessions, working memory training task and inhibition training task via the game *Braingame Brian*. Control: care as usual. Duration: 6 weeks. Assessment: baseline, posttest, 8 weeks, 12 weeks.	Inhibition Working memory	N: Training: 22, Control: 22. Mean age: Training: 11.5, Control: 11.41. Female (%): 50.	Inclusion: primary obesity, age 9–14, an IQ within the normal range, attend inpatient treatment program. Exclusion: have pervasive development disorders.	Overweight; mean adjusted BMI: Training: 131.58, Control: 132.91.	3

*NA, not available; BMI, body mass index. Bold values report significant results, as p-values <0.05*.

Eleven studies (Nederkoorn et al., 2007; Best et al., 2012; Spitznagel et al., 2013a,b, 2014; Kulendran et al., 2014; Galioto et al., 2016, 2018; Augustijn et al., 2018; Dassen et al., 2018a; Mackey et al., 2018) with 689 participants investigating whether baseline EFs can predict more weight loss in weight loss intervention were found. Several studies used one effect size to estimate more than one EF domain, and we classified these measures as general executive function. One study (Galioto et al., 2016) did not report non-significant results. Thus, 4 studies focused on general EF, 6 studies focused on inhibition, 4 studies focused on working memory, 5 studies focused on cognitive flexibility, 3 studies focused on delay discounting, and 2 studies focused on planning. The quality of studies was moderate to high except for one study that scored 5 points. The characteristics of the studies are summarized in Table 4.

**Table 4:** Longitudinal studies investigating whether better baseline EFs can predict greater weight loss through intervention.

**References**	**Intervention**	**Executive functions**	**Task(s) used**	**Participants**	**Weigh status**	**Time**	**Quality** **score**
Augustijn et al. (2018)	Multidisciplinary treatment program	Cognitive flexibility Planning General executive function	Cambridge Neuropsychological Test Automated Battery: IED (attention shifting), RVP (inhibition, updating), SOC (planning)	N: 32 Mean age: 9.6 Female (%): 56.3	Obesity. Mean BMI: 30.69. Mean body fat (%): 43.71.	5 months	7
Best et al. (2012)	Family-based weight-loss treatment	Delay discounting	Self-reported measures	N: 241 Mean age: 9.9 Female (%): 62.7	Overweight. Child percent obesity: 66.0.	4 months	7
Spitznagel et al. (2013a)	Bariatric surgery	General executive functions	The IntegNeuro cognitive test battery: digit span backward, switching of attention, verbal interference, letter fluency, maze task, verbal list-learning.	N: 57 Mean age: 43.65 Female (%): 87.7	Obesity. BMI: 46.49.	24 months	8
Dassen et al. (2018a)	Multidisciplinary weight loss program	Working memory Inhibition Delay discounting Cognitive flexibility	2-back task Stop-signal task Trial-making test Monetary Choice Questionnaire	N: 82 Weight status: Mean age: 42.12 Female (%): 74.4	Obesity. BMI: 38.94.	6 months	8
Galioto et al. (2016)	Medical weight loss program	Inhibition Cognitive flexibility	NIH EXAMINER battery: dot counting task, N-back test, flanker test, set shifting task, unstructured Task	N: 23 Mean age: 50.35 Female (%): 68	Obesity. BMI: 44.21.	2 months	6
Galioto et al. (2018)	Medical weight loss program	Working memory Inhibition Cognitive flexibility Planning	Behavior Rating Inventory of Executive Function-adult	N: 24 Mean age: 49.1 Female (%): 66.7	Obesity, BMI: 42.8.	2 months	6
Kulendran et al. (2014)	Multidimensional weight loss intervention	Inhibition Delay discounting	Stop-signal task Delay discounting task	N: 53 Mean age: 14.28 Female (%): 60.3	Obesity, overweight, normal weight, BMI: 33.75	2 months	8
Mackey et al. (2018)	Bariatric surgery	Inhibition Working memory	Tasks of executive control: N-back test	N: 12 Mean age: 17 Female (%): 58.3	Obesity, BMI: 48.5	6 months	8
Nederkoorn et al. (2007)	Behavior treatment	Inhibition	Stop-signal task	N: 26 Mean age: 9.3 Female (%): 65.4	Overweight. BMI: NA	14 months	5
Spitznagel et al. (2014)	Bariatric surgery	General executive functions	The IntegNeuro cognitive test battery: digit span backward, switching of attention, verbal interference, letter fluency, maze task, verbal list-learning.	N: 55 Mean age: 45 Female (%): 87.3	Obesity, BMI: 45.1	36 months	8
Spitznagel et al. (2013b)	Bariatric surgery	Working memory Cognitive flexibility General executive functions	The IntegNeuro cognitive test battery: switching of attention, verbal interference, maze task	N: 84 Mean age: 44.75 Female (%): 79.8	Obesity, BMI: 46.13	12 months	7

*NA, not available; BMI, body mass index*.

Six studies (Anzman and Birch, 2009; Nederkoorn et al., 2010; Schlam et al., 2013; Goldschmidt et al., 2015; Datar and Chung, 2018; Stinson et al., 2018) with 9988 participants investigating whether EF early in life can predict future weight loss were found. One study (Goldschmidt et al., 2015) used the Mazes test to measure planning. In fact, this test also involves other EFs, and therefore, we classified the measure as general EF. Four studies focused on inhibition, 1 study focused on general EF and 1 study focused on delay of gratification. One study (Stinson et al., 2018) did not report non-significant results. More information about the studies is shown in Table 5. The quality of the studies was moderate to high. We did not conduct a meta-analysis for general EF and delay gratification, as there was only one effect size for these two EF domains.

**Table 5:** Longitudinal studies investigating whether early life EFs can predict future weight loss.

**References**	***N***	**Mean age**	**Female (%)**	**Weight status**	**Executive functions**	**Task(s) used**	**Time**	**Quality score**
Anzman and Birch (2009)	197	7	100	BMI percentile: 59.7	Inhibition	The child behavior questionnaire (scale 1 to 7)	8 years	7
Datar and Chung (2018)	7,060	6	48.1	BMI: 16.5	Inhibition	Social skill rating system	8 years	8
Goldschmidt et al. (2015)	2,450	10	100	Obesity, overweight, normal weight. Normal weight BMI: 17.26 Obese or overweight BMI: 25.2	General EF	Wechsler intelligence scale for children-third edition, revised	6 years	8
Nederkoorn et al. (2010)	71	19.7	100	Mean BMI: 21.5	Inhibition	Stop-signal task	1 year	7
Schlam et al. (2013)	164	4	58	NA	Delay of gratification	Delay of gratification task	30 years	6
Stinson et al. (2018)	46	37.2	23.9	Mean BMI: 28.3	Inhibition	Stroop word color task	6 months to 3 years	7

*NA, not available; BMI, body mass index*.

### Meta-Analysis of RCTs

The results showed non-significant effects of general EF, inhibition, working memory, and cognitive flexibility, which indicated that the EF interventions were no more effective than the control conditions. The results are summarized in Table 6. A non-significant result of the Q-statistic for general EF, inhibition, working memory, and cognitive flexibility suggested that there existed a relatively good homogeneity among the studies. For general EF, the distribution of the variance across the three levels was as follows: level 1 was 100% (sampling variance), and level 2 (within-study variance) and level 3 (between-study variance) were 0%. For inhibition, the distribution of the variance across the three levels was as follows: level 1 was 100% (sampling variance), and level 2 (within-study variance) and level 3 (between-study variance) were 0%. The same pattern of the variance distribution was found for working memory and cognitive flexibility.

**Table 6:** Results from RCTs.

**EF domain**	**Effect size**		**Heterogeneity**				**Publication bias**	
	***g* (95% CI)**	***p***	**$I_{\text{within}}^{2}$**	**$I_{\text{between}}^{2}$**	***Q***	***p***	**Egger's test**	***p***
General EF	0.11 (−0.14–0.36)	0.31	0	0	1.04	0.96	0.92	0.4
Inhibition	−0.04 (−0.21–0.14)	0.66	0	0	5.02	0.93	−1.04	0.62
Working memory	0.05 (−0.27–0.37)	0.7	0	0	0.78	0.94	–**4.64**	**0.04**
Cognitive flexibility	0.12 (−0.27–0.52)	0.39	0	0	0.14	0.99	0.11	0.98

*Bold values report significant results, as p-values <0.05*.

We divided studies according to participant age (young adults: age 18 to 22; adults: age above 22) and mean baseline BMI (normal weight: BMI < 25; overweight: 25 ≤ BMI ≤ 30; obesity: BMI> 30). For inhibition, subgroup analyses of BMI (normal weight and obesity) and age (young adults and adults) were conducted. A non-significant overall effect size was found for obesity (*g* = −0.006, *p* = 0.947) and normal weight (*g* = 0.287, *p* = 0.221), and there was no significant difference between these two subgroups (*F* = 1.703, *p* = 0.221). The results for age were the same as those for BMI because the studies included in the subgroup analyses were the same. We did not conduct subgroup analysis for general EF, working memory, and cognitive flexibility, as the number of studies was <3.

**Figure 2:**
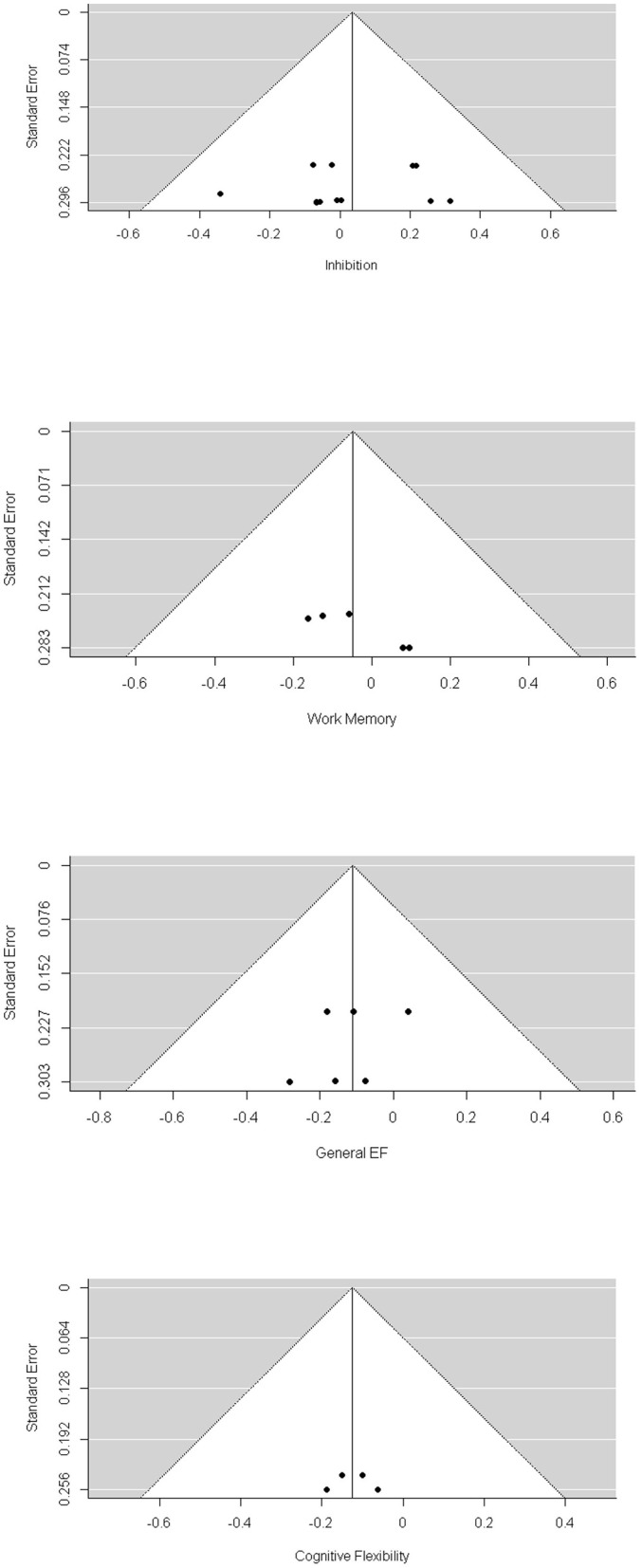
Funnel Plot of RCTs.

Visual inspection of the funnel plots (Figure 2) did not suggest that publication biases existed for general EF, inhibition, and cognitive flexibility. Egger's test also showed a non-significant result for general EF, inhibition, and cognitive flexibility. We found a significant publication bias for working memory (−4.635, *p* = 0.039). The trim-and-fill analysis indicated that there was one missing effect size for working memory, and the pooled effect size was still non-significant (*g* = 0.076, *p* = 0.47).

### Meta-Analysis of Longitudinal Studies Investigating Whether Baseline EFs Can Predict Greater Weight Loss Through Intervention

The results showed that better baseline inhibition significantly predicted more weight loss through weight loss treatment (β = 0.259, *p* = 0.03, 95% CI: 0.027–0.492), and better baseline delay discounting significantly predicted less weight loss (β= −0.17, *p* = 0.04, 95% CI: −0.331 to −0.011). The results showed that general EF, working memory, cognitive flexibility, and planning were not significant predictors. The results are summarized in Table 7. In terms of heterogeneity, non-significant Q statistics were found for general EF, cognitive flexibility, and delay discounting. We found significant Q statistics for inhibition (*Q* = 46.068, *p* < 0.0001), working memory (*Q* = 27.928, *p* = 0.002), and planning (*Q* = 6.838, *p* = 0.03). For general EF, the distribution of variance was 76.71% (level 1), 0% (level 2), and 23.29% (level 3). For inhibition, the distribution of variance was 4.89% (level 1), 95.11% (level 2), and 0% (level 3). For working memory, the distribution of variance was 19.94% (level 1), 72.58% (level 2), and 7.48% (level 3). For cognitive flexibility, the distribution of variance was 100% (level 1) and 0% for level 2 and level 3. For delay discounting, the distribution of variance was 100% (level 1) and 0% for level 2 and level 3. For planning, the distribution of variance was 24.93% (level 1), 75.07% (level 2), and 0% (level 3).

**Table 7:** Results from longitudinal studies investigating whether baseline EFs can predict greater weight loss through intervention.

**EF domain**	**Effect size**		**Heterogeneity**				**Publication bias**	
	**β (95% CI)**	***p***	**$I_{\text{within}}^{2}$**	**$I_{\text{between}}^{2}$**	***Q***	***p***	**Egger's test**	***p***
General EF	0.06 (−0.13–0.24)	0.45	0	23.29%	5.75	0.33	−0.07	0.96
Inhibition	**0.26 (0.03–0.49)**	**0.03**	95.11%	0	**46.07**	**<0.01**	**1.54**	**0.02**
Working memory	0.19 (−0.15–0.53)	0.23	72.58%	7.48%	**27.93**	**<0.01**	1.14	0.12
Cognitive flexibility	0 (−0.01–0.01)	0.38	0	0	9.16	0.42	−0.34	0.68
Delay discounting	−**0.17 (**−**0.33–**−**0.01)**	**0.04**	0	0	0.83	0.84	−0.42	0.54
Planning	0.17 (−0.46–0.8)	0.36	75.07%	0	**6.84**	**0.03**	2.68	0.06

*Bold values report significant results, as p-values < 0.05*.

We divided studies according to participant mean ages (children: age 7–11; adolescents age 12–17; young adults: age 18–22; adults: age above 22) and type of weight loss intervention (surgery and non-surgery). For general EF, subgroup analysis was conducted for age (children and adults) and intervention. Because the studies in subgroups in terms of age and intervention were the same, the results were also the same. We did not find moderating effects of age and intervention (*F* = 0.104, *p* = 0.76). For the children and non-surgery subgroups, the overall effect size was β = 0.09, *p* = 0.53; for the adults and surgery subgroups, the overall effect size was β = 0.04, *p* = 0.76. For inhibition, subgroup analysis was conducted for age (children, adolescents, and adults) and intervention (surgery and non-surgery). We found that age was not a moderating factor (*F* = 0.815, *p* = 0.47), and the overall effect size with children (β = 0.039, *p* = 0.51), adolescents (β = 0.459, *p* = 0.43), and adults (β = 0.241, *p* = 0.15) were non-significant. Intervention type (*F* = 2.39, *p* = 0.15) was also not a moderating factor. The overall effect size with non-surgery was marginally significant (β = 0.198, *p* = 0.07), while that with surgery was non-significant (β = 0.648, *p* = 0.15). For working memory, subgroup analysis was conducted for age (adolescents and adults) and intervention. We found a significant moderating effect of age (*F* = 13.666, *p* = 0.005) and significant overall effect size with both adolescents (β = 0.661, *p* = 0.002) and adults (β = 0.04, *p* = 0.005). The intervention type was not a moderating factor (*F* = 0.197, *p* = 0.67), and the overall effect sizes with surgery (β = 0.278, *p* = 0.67) and non-surgery (β = 0.116, *p* = 0.66) were not significant. We also found that the Q statistic for residual heterogeneity was not significant (*Q* = 12.417, *p* = 0.19). For cognitive flexibility, subgroup analysis was conducted for age (children and adults) and intervention. We did not find moderating effects of age (*F* = 0.185, *p* = 0.68) or intervention (*F* = 0.49, *p* = 0.5). The overall effect sizes with children (β = 0.004, *p* = 0.93), adults (β = −0.025, *p* = 0.68), surgery (β = 0.004, *p* = 0.37), and non-surgery (β = −0.046, *p* = 0.5) were not significant. There were only 3 studies focused on delay discounting; however, the mean participants' age of these studies varied from children, adolescents, and adults. Thus, we divided them into two subgroups: adults and non-adults. The result revealed a non-significant moderating effect (*F* = 0.42, *p* = 0.58). The overall effect size with non-adults was marginally significant (β = 0.186, *p* = 0.08), and the overall effect size with adults was non-significant (β = 0.1, *p* = 0.58). We did not conduct subgroup analysis for planning, as there were only two studies focused on this EF domain.

**Figure 3:**
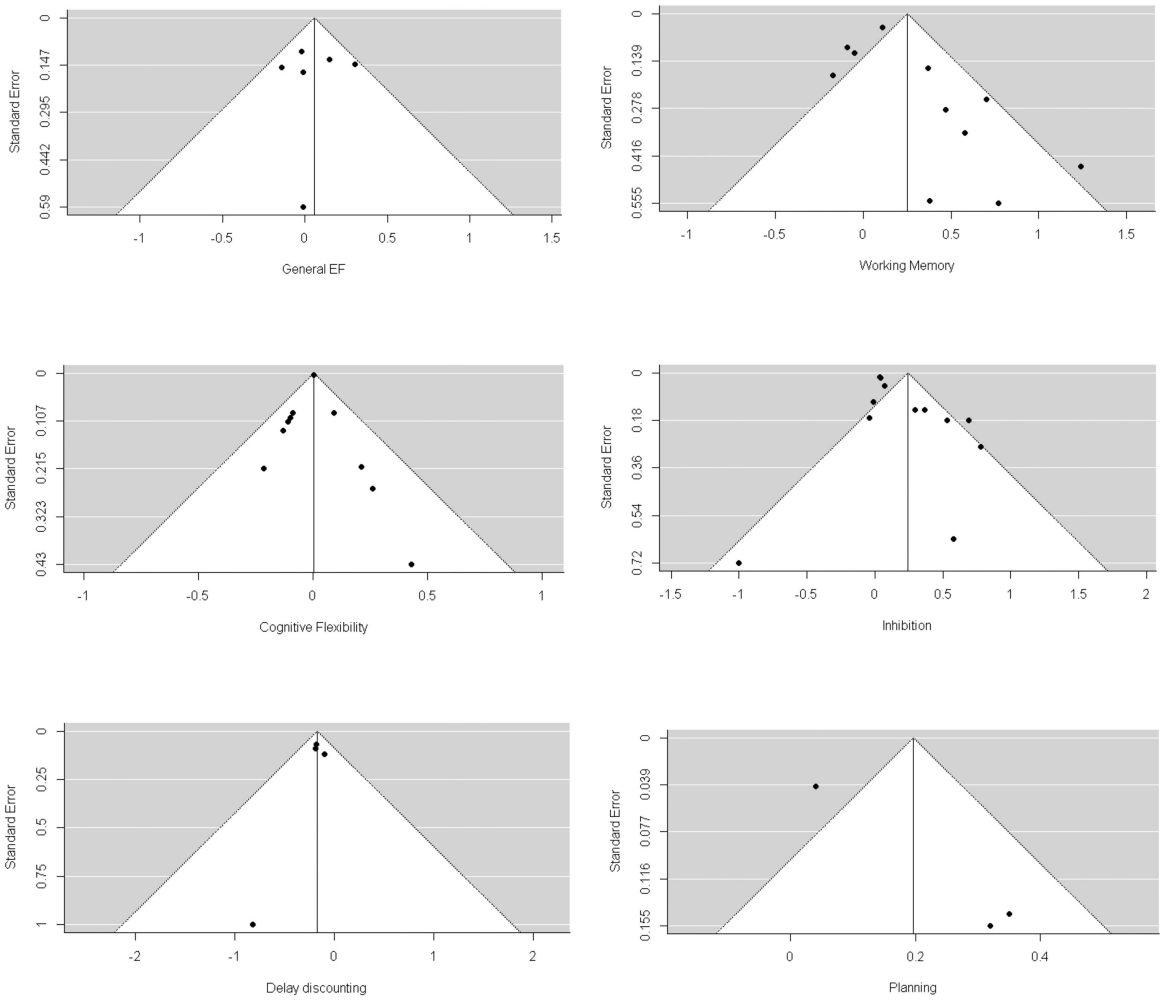
Funnel plots of longitudinal studies investigating whether baseline EFs can predict greater weight loss through intervention.

Through visual inspection of the funnel plot (Figure 3) and Egger's test, we found that general EF, working memory, cognitive flexibility, delay discounting, and planning were non-significant in terms of publication bias. However, we found a significant publication bias for inhibition (1.537, *p* = 0.02). The trim-and-fill analysis indicated that there were two missing effect sizes for inhibition, and the overall effect size of inhibition was still significant (β = 0.104, *p* = 0.03).

### Meta-Analysis of Longitudinal Studies Investigating Whether Early EFs Can Predict Future Weight Loss

We performed a multilevel meta-analysis only for inhibition, as there was only one study focused on delay of gratification and one on general EF. For delay of gratification, the effect size was 0.21; for general EF, the effect size was 0.05. The overall effect of inhibition was marginally significant (β = 0.185, *p* = 0.07, 95% CI: −0.021–0.391). A significant Q statistic result (*Q* = 25.364, *p* = 0.0001) indicated heterogeneity between studies. The distribution of variance was 12.74% (sampling variance), 80.93% (within-study variance), and 6.33% (between-study variance).

We divided studies according to the percentage of females in the studies (100% and not 100%) and age (children: age under 7; young adults: age 18–22; adults age above 22). We did not find a significant moderating effect of age (*F* = 0.027, *p* = 0.97) or gender (*F* = 0.005, *p* = 0.95). The overall effects with children (β = 0.179, *p* = 0.83), young adults (β = 0.19, *p* = 0.88), adults (β = 0.248, *p* = 0.38), 100% females (β = 0.194, *p* = 0.15), and non-100% females (β = 0.187, *p* = 0.95) were not significant.

**Figure 4:**
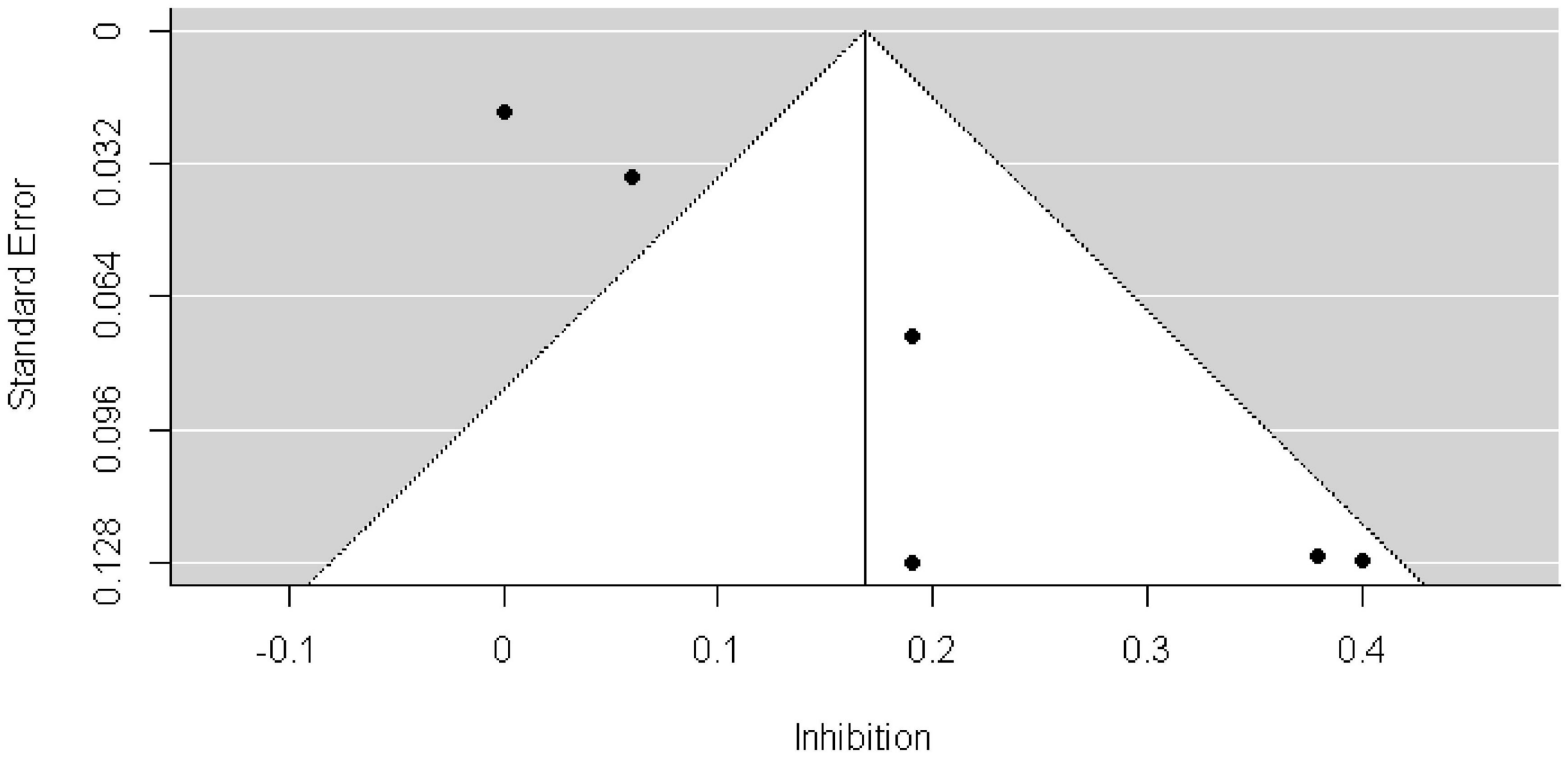
Funnel plots of longitudinal investigating whether early EFs can predict future weight status change.

We found a significant publication bias by Egger's test (3.129, *p* = 0.002) and by visual inspection of the funnel plot (Figure 4). Because the result of Egger's test was significant, we conducted the trill-and-fill analysis. The trim-and-fill analysis indicated that there were 3 missing effect sizes, and the overall effect size was not significant (β = 0.066, *p* = 0.19).

## Discussion

This is the first meta-analysis to investigate whether EF interventions have an effect on weight loss and whether EF can predict obesity intervention outcomes or future weight loss. Generally speaking, we found that (a) EF interventions have no effect on weight loss; (b) several baseline EF, such as inhibition and delay discounting, can predict obesity intervention outcomes; and (c) early-life inhibition can predict future weight loss. Our result may support the view that a higher-order factor, such as gene, may link obesity and EF together.

We found that EF interventions showed non-significant results on weight loss, even when publication bias was taken into consideration. This surprising result may suggest that EF has no direct effect on obesity or weight loss. The RCTs included in our meta-analysis mainly focused on changing participants' attitudes or behaviors toward foods by improving their EF. Researchers have reached a consensus that cognitive training has a significant effect on eat behaviors (Yang et al., 2019). Several studies included in our meta-analysis also reported that participants significantly changed their attitude and behavior toward food, while their BMI did not change (Stice et al., 2015; Dassen et al., 2018b). It is apparent that people with healthier eating behaviors have a greater possibility of weight loss. However, a non-significant result with the RCT studies seems to not support the assumption that EF can lead to obesity. This is a puzzling result. Favieri et al. (2019) argued that it is the bidirectional relationship between EF and obesity that causes the failure of the intervention. If this is true, obesity may hinder improvements in EF. However, most RCTs showed significant improvement in EF, which seems to not support his argument.

Consider that individual weight loss depends on differences in energy intake and expenditure; it is possible that participants in these RCTs reduced their energy intake as well as their energy expenditure, ultimately rendering EF interventions ineffective. As we discussed earlier, homeostasis, reward system, and EF are related to obesity. After EF training, obese individuals can resist food temptations effectively. However, they cannot alter their homeostasis system, and the homeostasis system begins to function so that the individual's energy expenditure decreases. The link between EF and obesity may be more complex than we thought.

One plausible explanation is that the relationship between EF and obesity may not be direct. The relationships between EF or eating behavior and weight status may not be as close as we think. There is no causal relationship between EF and obesity. A higher-order factor, such as gene, may link obesity and EF together, although such an assumption needs further research. Previous reviews of twins studies showed that 50–90% of BMI variance can be attributed to genes (Maes et al., 1997; Schousboe et al., 2003). Several genes were found to be the common genetic backgrounds of BMI and EF (Bressler et al., 2013; Zhao et al., 2013; Marioni et al., 2016). This hypothesis of the relationship between EF and obesity increases our understanding of the mechanisms of obesity. Since obesity and EF have a common genetic background, obesity could not lead to poor EF, and poor EF could not result in obesity, it is likely that they are accompanying phenomena. An individual's obesity and poor EF may occur together. Poor EF and obesity may interact in a variety of ways, creating a vicious cycle (Tomiyama, 2019).

Another explanation of such a result is that the indicator of weight status is not suitable. All the included studies used BMI as the indicator, and only a few studies employed other indicators, such as body fat and waist circumference, in addition to BMI. As many researchers have argued, BMI cannot distinguish the centralized distribution of visceral fat or central obesity and may not explain obesity-related health risk (Micozzi and Albanes, 1987; Janssen et al., 2004). Notably, a non-significant result does not mean that there was no effect, especially when the number of studies included in the meta-analysis was too small.

We found that baseline inhibition and delay discounting were significant predictors of weight loss through intervention, which was in agreement with other empirical studies (Weller et al., 2008; Epstein et al., 2010). This result could also be interpreted by the assumption that the relationship between EF and obesity is not direct. People with better EF may be indicative of a genetic background that allows them to lose more weight. The result of delay discounting is consistent with the result that the ability to delay gratification at age 4 can predict lower BMI 30 years later (Schlam et al., 2013), as delay discounting is reverse of delay of gratification (Anokhin et al., 2011). Despite the significant predictive effect of delay discounting, obesity intervention through reductions in delay discounting, which may be a promising treatment, has not yet been proposed.

Accordingly, we found that early-life inhibition significantly predicted future weight loss. There was significant heterogeneity between studies. When taking publication bias into consideration, we obtained a non-significant result. However, this approach may not be suitable for testing publication bias because one assumption of publication tests is homogeneity of the data. If this assumption is violated, publication bias tests cannot distinguish heterogeneity and publication bias and might lead to uninterpretable results (Ioannidis, 2005). The significant predictive effect of inhibition and delay discounting on weight loss through both obesity interventions and natural conditions indicates that such a relationship is relatively stable. Therefore, EF is a good predictor of weight loss and obesity, and EF training in early life may be helpful to prevent obesity. Limited by the number of studies, we did not investigate whether other types of EFs can predict future weight loss.

We found that although baseline working memory was not a significant predictor of weight loss through intervention, the subgroup analysis showed that age played a moderating role, and the results of the two subgroups (adolescents and adults) were significant which indicated that baseline working memory did have an effect on obesity intervention outcomes. Except for age, we did not find any other moderating factors. A non-significant moderating effect of age in other EF domains may be attributed to the different developmental trajectories of EFs. However, there exists a conflict regarding age as a moderator in other meta-analyses investigating the relationship between EF and obesity. Yang et al. (2018) found that age was not a moderator of the relationship between obesity and EF, while Wu et al. (2015) showed that age was a moderator. One explanation of these conflicting data is, as Yang et al. (2018) argued, due to the different analytic strategies. This may also be due to the type of study. In our study, we wanted to investigate whether EF can influence obesity, while Yang et al. (2018) and Wu et al. (2015) investigated whether there were differences in EF performances between obese and normal-weight individuals. Empirical studies have also reported these conflicting results. Gunstad et al. (2007) found that the relationship between obesity and executive dysfunction was not associated with age. In contrast, the brain activation measured by functional near-infrared spectroscopy technology (fNIRS) due to Stroop interference did not increase with age in obese subjects (Huang et al., 2019), while activation in normal-weight individuals continued to increase (Adleman et al., 2002). The role of age needs further study.

## Limitations and Future Directions

There are several limitations in the current study. First, as the number of studies included in the meta-analysis was small, the results we obtained may be biased. Thus, more RCTs investigating whether EF can impact obesity and more longitudinal studies investigating whether EF can predict weight loss through obesity intervention or in natural conditions are needed. Second, we did not investigate the role of the weight status indicator. As mentioned above, BMI may not be the appropriate indicator; it is possible that using different obesity indicators for the same population could lead different results. In fact, one RCT included in our meta-analysis found that after EF intervention, participants showed a significant body fat reduction while their BMI was not changed (Stice et al., 2017). To avoid the bias caused by obesity indicators, multiple indicators should be used in future studies. Third, although we investigated the role of gender, this was based on the proportion of females. We know that there are differences between males and females in obesity (Schousboe et al., 2003). The prevalence of obesity is different in men and women (Flegal et al., 2012). Moreover, obese men and women are different in brain activation (Mueller et al., 2011) and brain functional connection (Atalayer et al., 2014) when dealing with food cues. Gender plays an important role in obesity, and the role of gender in the relationship between obesity and EF needs to be further explored. Finally, the tools used to measure EF may not have been adequate. The EF tasks we used may not be pure. Although it has been stated that the tasks measure a specific EF, it is difficult to rule out the possibility that there are no other EFs involves in the tasks. In addition, the relationship between EF and frontal lobe activity may not be one-to-one (Alvarez and Emory, 2006). People with frontal lobe lesions may perform as well as normal individuals in a particular EF task (Ahola et al., 1996), and healthy people may perform as poor as individuals with frontal lobe lesions (Axelrod et al., 1996). With the development of neuroimaging technology, an increasing number of studies have used brain imaging data as indicator of EF (Kishinevsky et al., 2012; Hege et al., 2013), which is a promising area.

## Conclusion

This is a meta-analysis to investigate whether EFs have an impact on obesity and weight loss. Specifically, we investigate whether EF interventions have an effect on obesity and weight loss and whether baseline EF can predict future weight loss through obesity interventions and in natural conditions. We also examined some potential moderating factors, such as age, weight status, type of intervention, and percentage of female participants. First, the results showed that EF interventions may not have an effect on weight loss, which indicates that the relationship between EF and obesity may not be direct. A higher-order factor such as genes may link obesity and EF. However, as the number of studies included in the meta-analysis was small, the results we obtained may be biased, and more interventional studies are needed. Second, we found that baseline inhibition and delay discounting can significantly predict weight loss through obesity interventions and natural conditions, which shows that EF is a good predictor for weight loss and obesity. Therefore, EF training in early life may be an effective way to prevent obesity. Since EF can predict the outcome of obesity interventions, we may be able to provide more personalized treatment based on subjects' baseline EF. Despite the significant predictive effect of delay discounting, obesity intervention through reductions in delay discounting, which may be a promising treatment, has not yet been proposed. Finally, age moderates the relationship between working memory and weight loss through intervention, but not weight status, type of intervention, and percentage of female. Although we investigated several potential moderators, we cannot draw a strong conclusion based on the existing results. The roles of age, gender, and obesity indicators remain unclear and need to be further explored. In addition, neuroimaging technology may overcome the shortcomings of behavioral researches, which is a promising area.

## Data Availability Statement

The original contributions presented in the study are included in the article/supplementary material, further inquiries can be directed to the corresponding author/s.

## Author Contributions

ZD conceived and designed the study, collected and interpreted the data, and wrote the first draft of the manuscript. JL reviewed the manuscript. JH modified the manuscript. XiaoX and JM collected and interpreted the data. RZ interpreted the data. XiaX conceived, designed the study and modified, and reviewed the manuscript. All authors critically revised the manuscript and approved the final version.

## Conflict of Interest

The authors declare that the research was conducted in the absence of any commercial or financial relationships that could be construed as a potential conflict of interest.
